# Diaqua­bis­(1*H*-imidazole-4-carboxyl­ato-κ^2^ *N* ^3^,*O*)zinc

**DOI:** 10.1107/S1600536811020800

**Published:** 2011-06-11

**Authors:** Wenlin Shuai, Songliang Cai, Shengrun Zheng

**Affiliations:** a Guangdong Test Center for Green Labelling, Guangzhou 510440, People’s Republic of China; b School of Chemistry and Environment, South China Normal University, Guangzhou 510006, People’s Republic of China

## Abstract

In the title compound, [Zn(C_4_H_3_N_2_O_2_)_2_(H_2_O)_2_], the Zn^II^ ion is situated on a twofold rotation axis and exhibits a distorted octa­hedral coordination configuration. The equatorial plane contains two *cis*-oriented bidentate 1*H*-imidazole-4-carboxyl­ate ligands and the axial positions are occupied by two coordinated water mol­ecules. In the crystal structure, inter­molecular O—H⋯O and N—H⋯O hydrogen bonds link the mol­ecules into a three-dimensional supra­molecular network. There are π–π inter­actions between the imidazole rings, with a centroid-to-centroid distance of 3.504 (3) Å.

## Related literature

For general background, see: Yin *et al.* (2009 ▶); Zheng *et al.* (2011) ▶; Alkordi *et al.* (2009 ▶); Lu *et al.* (2009 ▶). For related structures, see: Haggag (2005 ▶); Starosta & Leciejewicz (2006 ▶); Gryz *et al.* (2007 ▶); Yin *et al.* (2009 ▶).
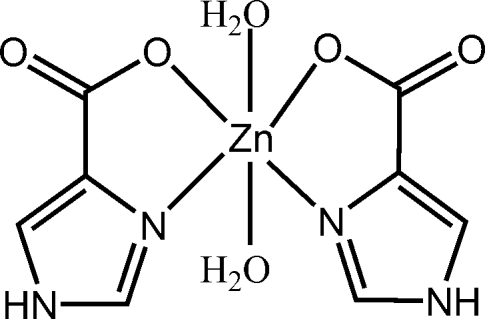


## Experimental

### 

#### Crystal data

[Zn(C_4_H_3_N_2_O_2_)_2_(H_2_O)_2_]
*M* *_r_* = 323.57
Orthorhombic, 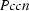

*a* = 7.1399 (19) Å
*b* = 11.757 (3) Å
*c* = 13.508 (4) Å
*V* = 1133.9 (5) Å^3^
*Z* = 4
Mo *K*α radiation
μ = 2.20 mm^−1^
*T* = 298 K
0.35 × 0.32 × 0.30 mm

#### Data collection

Bruker SMART APEXII CCD area-detector diffractometer
Absorption correction: multi-scan (*SADABS*; Sheldrick, 1996 ▶) *T*_min_ = 0.513, *T*_max_ = 0.558
5336 measured reflections
1037 independent reflections
913 reflections with *I* > 2σ(*I*)
*R*_int_ = 0.021

#### Refinement

*R*[*F* ^2^ > 2σ(*F* ^2^)] = 0.023
*wR*(*F* ^2^) = 0.066
*S* = 1.08
1037 reflections
95 parameters
2 restraints
H atoms treated by a mixture of independent and constrained refinement
Δρ_max_ = 0.29 e Å^−3^
Δρ_min_ = −0.33 e Å^−3^

### 

Data collection: *APEX2* (Bruker, 2004 ▶); cell refinement: *APEX2*; data reduction: *APEX2*; program(s) used to solve structure: *SHELXS97* (Sheldrick, 2008 ▶); program(s) used to refine structure: *SHELXL97* (Sheldrick, 2008 ▶); molecular graphics: *XP* in *SHELXTL* (Sheldrick, 2008 ▶); software used to prepare material for publication: *SHELXTL*.

## Supplementary Material

Crystal structure: contains datablock(s) I, global. DOI: 10.1107/S1600536811020800/cv5098sup1.cif

Structure factors: contains datablock(s) I. DOI: 10.1107/S1600536811020800/cv5098Isup2.hkl

Additional supplementary materials:  crystallographic information; 3D view; checkCIF report

## Figures and Tables

**Table 1:** Hydrogen-bond geometry (Å, °)

*D*—H⋯*A*	*D*—H	H⋯*A*	*D*⋯*A*	*D*—H⋯*A*
N2—H2⋯O2^i^	0.86	1.93	2.784 (2)	174
O1*W*—H1*WA*⋯O2^ii^	0.83 (2)	2.04 (2)	2.850 (2)	167 (3)
O1*W*—H1*WB*⋯O2^iii^	0.82 (2)	1.96 (2)	2.778 (2)	175 (3)

Symmetry codes: (i) 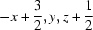; (ii) 
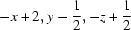
; (iii) 
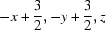
.

**Fig. 1.**
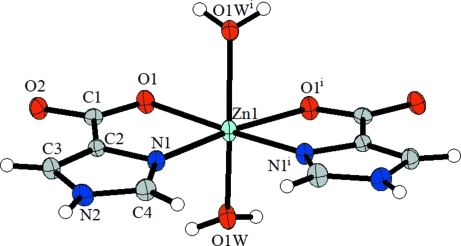
The molecular structure of (I), with displacement ellipsoids drawn at the 30% probability level [symmetry code: (i) -x + 5/2, -y + 3/2, z.]

**Fig. 2.**
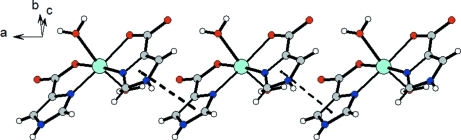
A portion of the crystal packing showing π–π interactions between the imidazole rings as dashed lines.

## supplementary crystallographic information

### Comment

Recently, we were interested in constructing coordination polymers based on *N*-heterocyclic carboxylic acids (Zheng *et al.*, 2011). The imidazole-4-carboxylatic acid (H_2_imc), which contains two N atoms of an imidazole group and one carboxylate group, remains largely unexplored, compared with its analogue imidazole-4,5-dicarboxylic acid (Alkordi *et al.*, 2009; Lu *et al.*, 2009). To date, only a few mononuclear complexes based on the H_2_imc ligand have been documented (Haggag, 2005; Starosta & Leciejewicz, 2006; Gryz *et al.*, 2007; Yin *et al.*, 2009). For instance, Yin *et al.* (2009) reported the structure of a mononuclear complex [Cd(Himc)_2_(H_2_O)_2_], which was prepared by the solvent evaporation method. Herein, we report a new Zn^II^ coordination polymer [Zn(Himc)_2_(H_2_O)_2_], (I), which is isomorphous with the Cd^II^ analog.

The asymmetric unit of (I) contains a half of [Zn(Himc)_2_(H_2_O)_2_] formula unit. The Zn^II^ ion exhibits a distorted octahedral geometry (Fig. 1), in which two *cis*-oriented bidentate chelating Himc^-^ ligands are located in the equatorial plane, forming two stable five-membered rings with metal ion, and the axial sites are occupied by two coordinated water molecules (Fig. 1). The Zn—O distances range from 2.1623 (17) to 2.1626 (14) Å and Zn—N bonds have the value of 2.0751 (16) Å. All Zn—O and Zn—N bond distances are shorter than those of Cd^II^ analog [the axial Cd—O, the equatorial Cd—O and Cd—N bond distances are 2.343 (2), 2.325 (2) and 2.274 (2) Å, respectively].

In the crystal structure, intermolecular O—H···O and N—H···O hydrogen bonds (Table 1) link the molecules into a three-dimensional supramolecular network, which demonstrate π–π interactions between the imidazole rings (Fig. 2) with the centroid-to-centroid distance of 3.504 (3) Å.

### Experimental

13.6 mg ZnCl_2_ (0.10 mmol) and 16.8 mg H_2_imc (0.20 mmol) were mixed in 6 ml EtOH/H_2_O (1:1). The aqueous NaOH (0.20 *M*) solution was dropwise added to the above solution and the pH was adjusted to about 7. Then, the resulting mixture was sealed into a 10 ml Teflon-lined stainless-steel reactor, which was heated at 100°C for 48 h under autogenous pressure, and then slowly cooled to room temperature at a rate of 2°C/h. Colorless block crystals of (I) were isolated, washed with distilled water, and dried in air (yield: 56%). IR (KBr, n/cm^-1^): 3382*m*, 3147 s, 2997w, 2941w, 2849w, 1688*m*, 1584 s, 15581*m*, 1462*m*, 1402w, 1395 s, 1237 s, 1094*m*, 1003 s, 931w, 847w, 820w, 793*m*, 713w, 656*m*, 610w, 494*m*.

### Refinement

C- and N-bound H atoms were positioned geometrically and refined using a riding model, with C—H = 0.93 and N—H = 0.86 Å and with *U*_iso_(H) = 1.2U_eq_(C,N). H atoms of the water molecule were located from a difference Fourier map and isotropically refined with O—H bond lenghts restrained to 0.82 (2) Å.

### Crystal data

**Table:**

[Zn(C_4_H_3_N_2_O_2_)_2_(H_2_O)_2_]	*D*_x_ = 1.895 Mg m^−^^3^
*M**_r_* = 323.57	Mo *K*α radiation, λ = 0.71073 Å
Orthorhombic, *P**c**c**n*	Cell parameters from 2272 reflections
*a* = 7.1399 (19) Å	θ = 3.3–27.3°
*b* = 11.757 (3) Å	µ = 2.20 mm^−^^1^
*c* = 13.508 (4) Å	*T* = 298 K
*V* = 1133.9 (5) Å^3^	Block, colourless
*Z* = 4	0.35 × 0.32 × 0.30 mm
*F*(000) = 656	

### Data collection

**Table:**

Bruker SMART APEXII CCD area-detector diffractometer	1037 independent reflections
Radiation source: fine-focus sealed tube	913 reflections with *I* > 2σ(*I*)
graphite	*R*_int_ = 0.021
φ and ω scans	θ_max_ = 25.2°, θ_min_ = 3.0°
Absorption correction: multi-scan (*SADABS*; Sheldrick, 1996)	*h* = −7→8
*T*_min_ = 0.513, *T*_max_ = 0.558	*k* = −8→14
5336 measured reflections	*l* = −14→16

### Refinement

**Table:**

Refinement on *F*^2^	Primary atom site location: structure-invariant direct methods
Least-squares matrix: full	Secondary atom site location: difference Fourier map
*R*[*F*^2^ > 2σ(*F*^2^)] = 0.023	Hydrogen site location: inferred from neighbouring sites
*wR*(*F*^2^) = 0.066	H atoms treated by a mixture of independent and constrained refinement
*S* = 1.08	*w* = 1/[σ^2^(*F*_o_^2^) + (0.0338*P*)^2^ + 0.618*P*] where *P* = (*F*_o_^2^ + 2*F*_c_^2^)/3
1037 reflections	(Δ/σ)_max_ < 0.001
95 parameters	Δρ_max_ = 0.29 e Å^−^^3^
2 restraints	Δρ_min_ = −0.33 e Å^−^^3^

### Special details

**Table:**

Geometry. All e.s.d.'s (except the e.s.d. in the dihedral angle between two l.s. planes) are estimated using the full covariance matrix. The cell e.s.d.'s are taken into account individually in the estimation of e.s.d.'s in distances, angles and torsion angles; correlations between e.s.d.'s in cell parameters are only used when they are defined by crystal symmetry. An approximate (isotropic) treatment of cell e.s.d.'s is used for estimating e.s.d.'s involving l.s. planes.
Refinement. Refinement of *F*^2^ against ALL reflections. The weighted *R*-factor *wR* and goodness of fit *S* are based on *F*^2^, conventional *R*-factors *R* are based on *F*, with *F* set to zero for negative *F*^2^. The threshold expression of *F*^2^ > σ(*F*^2^) is used only for calculating *R*-factors(gt) *etc*. and is not relevant to the choice of reflections for refinement. *R*-factors based on *F*^2^ are statistically about twice as large as those based on *F*, and *R*-factors based on ALL data will be even larger.

### Fractional atomic coordinates and isotropic or equivalent isotropic displacement parameters (Å^2^)

**Table:**

	*x*	*y*	*z*	*U*_iso_*/*U*_eq_	
Zn1	1.2500	0.7500	0.37215 (2)	0.02665 (15)	
N1	1.0565 (2)	0.81059 (14)	0.47393 (11)	0.0263 (4)	
N2	0.8608 (2)	0.87069 (15)	0.58685 (12)	0.0318 (4)	
H2	0.8123	0.8836	0.6440	0.038*	
C2	0.9100 (3)	0.86538 (16)	0.42860 (13)	0.0229 (4)	
C1	0.9084 (3)	0.87026 (16)	0.31884 (14)	0.0229 (4)	
C3	0.7885 (3)	0.90319 (19)	0.49823 (15)	0.0291 (4)	
H3	0.6781	0.9433	0.4875	0.035*	
C4	1.0207 (3)	0.81514 (18)	0.56937 (14)	0.0322 (5)	
H4	1.0966	0.7838	0.6182	0.039*	
O1	1.04760 (19)	0.82909 (12)	0.27495 (9)	0.0304 (3)	
O2	0.76821 (19)	0.91520 (13)	0.27745 (10)	0.0303 (4)	
O1W	1.0979 (2)	0.59370 (14)	0.34656 (12)	0.0356 (4)	
H1WA	1.150 (4)	0.550 (2)	0.3077 (18)	0.062 (9)*	
H1WB	0.989 (3)	0.595 (3)	0.328 (2)	0.072 (10)*	

### Atomic displacement parameters (Å^2^)

**Table:**

	*U*^11^	*U*^22^	*U*^33^	*U*^12^	*U*^13^	*U*^23^
Zn1	0.0242 (2)	0.0358 (2)	0.0200 (2)	0.00824 (13)	0.000	0.000
N1	0.0267 (9)	0.0336 (10)	0.0187 (8)	0.0059 (7)	0.0010 (7)	0.0021 (7)
N2	0.0338 (10)	0.0426 (11)	0.0189 (8)	0.0024 (8)	0.0070 (7)	−0.0012 (7)
C2	0.0220 (10)	0.0252 (10)	0.0214 (10)	0.0008 (8)	0.0003 (8)	0.0002 (8)
C1	0.0246 (10)	0.0238 (10)	0.0205 (9)	−0.0028 (8)	−0.0016 (8)	0.0009 (8)
C3	0.0254 (10)	0.0344 (11)	0.0275 (11)	0.0040 (9)	0.0007 (9)	−0.0018 (9)
C4	0.0344 (12)	0.0421 (13)	0.0203 (10)	0.0044 (9)	−0.0009 (9)	0.0046 (9)
O1	0.0283 (8)	0.0434 (9)	0.0194 (7)	0.0063 (6)	0.0021 (6)	−0.0011 (6)
O2	0.0249 (8)	0.0433 (9)	0.0227 (7)	0.0044 (6)	−0.0045 (6)	0.0051 (6)
O1W	0.0264 (8)	0.0386 (9)	0.0417 (9)	0.0049 (7)	−0.0033 (7)	−0.0108 (7)

### Geometric parameters (Å, °)

**Table:**

Zn1—N1	2.0751 (16)	N2—H2	0.8600
Zn1—N1^i^	2.0751 (16)	C2—C3	1.354 (3)
Zn1—O1	2.1626 (14)	C2—C1	1.484 (3)
Zn1—O1^i^	2.1626 (14)	C1—O1	1.255 (2)
Zn1—O1W^i^	2.1623 (17)	C1—O2	1.262 (2)
Zn1—O1W	2.1623 (17)	C3—H3	0.9300
N1—C4	1.315 (2)	C4—H4	0.9300
N1—C2	1.373 (2)	O1W—H1WA	0.828 (17)
N2—C4	1.336 (3)	O1W—H1WB	0.818 (18)
N2—C3	1.358 (3)		
			
N1—Zn1—N1^i^	97.01 (9)	C4—N2—H2	126.1
N1—Zn1—O1	79.04 (6)	C3—N2—H2	126.1
N1^i^—Zn1—O1	174.10 (5)	C3—C2—N1	109.40 (16)
N1—Zn1—O1^i^	174.10 (5)	C3—C2—C1	132.55 (17)
N1^i^—Zn1—O1^i^	79.04 (6)	N1—C2—C1	118.02 (15)
O1—Zn1—O1^i^	105.24 (7)	O1—C1—O2	125.47 (17)
N1—Zn1—O1W^i^	98.53 (7)	O1—C1—C2	116.81 (15)
N1^i^—Zn1—O1W^i^	93.64 (7)	O2—C1—C2	117.71 (16)
O1—Zn1—O1W^i^	82.71 (6)	C2—C3—N2	106.05 (18)
O1^i^—Zn1—O1W^i^	86.15 (6)	C2—C3—H3	127.0
N1—Zn1—O1W	93.64 (7)	N2—C3—H3	127.0
N1^i^—Zn1—O1W	98.54 (7)	N1—C4—N2	111.05 (18)
O1—Zn1—O1W	86.15 (6)	N1—C4—H4	124.5
O1^i^—Zn1—O1W	82.71 (6)	N2—C4—H4	124.5
O1W^i^—Zn1—O1W	161.60 (9)	C1—O1—Zn1	114.11 (11)
C4—N1—C2	105.65 (16)	Zn1—O1W—H1WA	114 (2)
C4—N1—Zn1	142.45 (15)	Zn1—O1W—H1WB	121 (2)
C2—N1—Zn1	111.89 (12)	H1WA—O1W—H1WB	104 (3)
C4—N2—C3	107.84 (17)		
			
N1^i^—Zn1—N1—C4	−5.0 (2)	C3—C2—C1—O2	−1.8 (3)
O1—Zn1—N1—C4	179.4 (3)	N1—C2—C1—O2	176.18 (18)
O1^i^—Zn1—N1—C4	42.6 (7)	N1—C2—C3—N2	−0.3 (2)
O1W^i^—Zn1—N1—C4	−99.8 (2)	C1—C2—C3—N2	177.80 (19)
O1W—Zn1—N1—C4	94.1 (3)	C4—N2—C3—C2	0.0 (2)
N1^i^—Zn1—N1—C2	175.82 (16)	C2—N1—C4—N2	−0.6 (2)
O1—Zn1—N1—C2	0.25 (13)	Zn1—N1—C4—N2	−179.84 (17)
O1^i^—Zn1—N1—C2	−136.6 (5)	C3—N2—C4—N1	0.4 (3)
O1W^i^—Zn1—N1—C2	81.05 (14)	O2—C1—O1—Zn1	−176.15 (15)
O1W—Zn1—N1—C2	−85.11 (14)	C2—C1—O1—Zn1	3.9 (2)
C4—N1—C2—C3	0.6 (2)	N1—Zn1—O1—C1	−2.39 (14)
Zn1—N1—C2—C3	−179.92 (14)	N1^i^—Zn1—O1—C1	−50.7 (6)
C4—N1—C2—C1	−177.86 (17)	O1^i^—Zn1—O1—C1	173.43 (15)
Zn1—N1—C2—C1	1.6 (2)	O1W^i^—Zn1—O1—C1	−102.60 (14)
C3—C2—C1—O1	178.1 (2)	O1W—Zn1—O1—C1	92.07 (14)
N1—C2—C1—O1	−3.9 (2)		

Symmetry codes: (i) −*x*+5/2, −*y*+3/2, *z*.

### Hydrogen-bond geometry (Å, °)

**Table:**

*D*—H···*A*	*D*—H	H···*A*	*D*···*A*	*D*—H···*A*
N2—H2···O2^ii^	0.86	1.93	2.784 (2)	174.
O1W—H1WA···O2^iii^	0.83 (2)	2.04 (2)	2.850 (2)	167 (3)
O1W—H1WB···O2^iv^	0.82 (2)	1.96 (2)	2.778 (2)	175 (3)

Symmetry codes: (ii) −*x*+3/2, *y*, *z*+1/2; (iii) −*x*+2, *y*−1/2, −*z*+1/2; (iv) −*x*+3/2, −*y*+3/2, *z*.
